# Wide-Angle Optical Metasurface for Vortex Beam Generation

**DOI:** 10.3390/nano13192680

**Published:** 2023-09-29

**Authors:** Meng-Hsin Chen, Bo-Wen Chen, Kai-Lun Xu, Vin-Cent Su

**Affiliations:** Department of Electrical Engineering, National United University, Miaoli 36003, Taiwan; mhc@nuu.edu.tw (M.-H.C.); m1021022@o365.nuu.edu.tw (B.-W.C.); m1021027@o365.nuu.edu.tw (K.-L.X.)

**Keywords:** metasurface, nanostructure, gallium nitride, optical vortex

## Abstract

In this work, we have achieved an advancement by integrating wide-angle capacity into vortex beams with an impressive topological charge (TC) of 12. This accomplishment was realized through the meticulous engineering of a propagation-phase-designed metasurface. Comprising gallium nitride (GaN), meta-structures characterized by their high-aspect ratio, this metasurface exhibits an average co-polarization transmission efficiency, reaching a remarkable simulated value of up to 97%. The intricate spiral patterns, along with their respective quantification, have been meticulously investigated through tilt-view scanning electron microscopy (SEM) and were further analyzed through the Mach–Zehnder interferometer. A captivating revelation emerged, a distinctive petal-like interference pattern manifests prior to the metasurface’s designed focal distance. The occurrence of this petal-like pattern at a specific *z*-axis position prompts a deliberate manipulation of the helicity of the spiral branches. This strategic helicity alteration is intrinsically tied to the achievement of a minimized donut diameter at the designed focal length. In regard to the angular capability of the device, the captured images continuously showcase prominent attributes within incident angles spanning up to 30 degrees. However, as incident angles surpass the 30-degree threshold, the measured values diverge from their corresponding theoretical projections, resulting in a progressive reduction in the completeness of the donut-shaped structure.

## 1. Introduction

As a ubiquitous phenomenon within optics, oblique incidence occurs when light rays intersect surfaces at an angle other than normal to the surface. This fundamental behavior holds significance across scientific and technological realms. Wide-angle optical components [1], born from harnessing the potential of oblique incident light, present a paradigm shift by endowing optical systems with panoramic capabilities. These components find versatile applications in domains such as astronomy, photography, and surveillance. They can emphasize foreground objects with a dramatic perspective while incorporating a significant portion of the background. Wide-angle lenses are characterized by their short focal lengths that capture more of the surroundings, resulting in a sense of depth and spaciousness in the captured image. However, conventional wide-angle lenses face challenges from stacking multiple optical components, yielding bulky and heavy optical devices. Moreover, these lenses are incompatible with compact and flat optical systems manufactured through CMOS-compatible semiconductor processes. This incongruence with evolving fabrication techniques calls for innovative solutions to realize the potential of wide-angle lenses in future optical landscapes.

The emergence of innovative metadevices [2,3] has captivated various research communities in recent decades, driven by their capacity to unlock optical phenomena unattainable in traditional optics. As a 2D category of metadevices, metasurfaces consisting of artificial meta-structures arranged in sub-wavelength periods adhere to the generalized laws of refraction and reflection [4]. By introducing these meta-structures at interfaces between media, an abrupt phase discontinuity emerges, ushering in anomalous behaviors of light reflection and refraction [4,5]. According to constituent materials for the meta-structures, plasmonic metasurfaces [6,7] have found extensive application in reflective scenarios. The counterpart, dielectric metasurfaces, has paved the way for applications of transmission operation encompassing metalenses [8,9,10,11,12], meta-holograms [13], optical vortices [14], and quantum optics [15]. In this series of accomplishments, the pursuit of wide-angle metalenses [16,17,18,19,20,21] has culminated in diffraction-limited focal spots. However, the exploration of intricate output wavefront shaping while harnessing wide-angle incidence remains an uncharted realm.

One of the most remarkable manifestations of complex wavefronts is in optical vortex beams [22], where a propagating optical vortex [23] carries orbital angular momentum (OAM) within the paraxial approximation. Such beams exhibit helical wavefronts, defined by a phase-dependent Hilbert factor represented as $e^{im\theta }$, where m denotes the OAM mode and θ signifies phase. This configuration engenders a unique donut-shaped light intensity distribution, featuring a phase singularity at the center and a 2πm phase variation azimuthally. Optical vortex beams carrying OAM possess profound significance owing to their versatile OAM mode selections that have found diverse applications, including optical communication [24,25], optical manipulation through optical tweezers [26], super-resolution imaging [27,28], and quantum technologies [29,30]. While conventional methods for vortex beam emission necessitate cumbersome components such as spiral phase plates [31], spatial light modulation [32,33], or diffractive gratings [34,35], optical metasurfaces have emerged as efficient platforms for their generation. Unfortunately, the existing optical metasurfaces tailored for vortex beam generation exhibit limitations in accommodating wide-angle incidence [36,37]. This limitation inevitably engenders complications in optimizing the coupling efficiency between light signals and optical fibers [38,39]. Notably, this challenge increases with the elevation of the OAM mode.

In this study, we present a successful demonstration of a wide-angle metasurface designed for the generation of optical vortex beams with a remarkably high OAM mode of 12. To engineer this intricate phase distribution essential for the construction of the metasurface, we have strategically opted for a high refractive-index contrast material system. This system leverages high-aspect-ratio meta-structures composed of gallium nitride (GaN) on a sapphire substrate, which was polished on both sides. The design principle based on the propagation phase was employed to precisely implement the required phase profile of the metasurface. Following a meticulous inspection conducted using an optical microscope (OM) and a scanning electron microscope (SEM), the device was subjected to the normal incidence measurement to ascertain the congruence between the observed optical vortex beam generations and the intended design. Additionally, a Mach–Zehnder interferometer was employed to rigorously probe the behavior of the optical vortex metasurface through interference measurements. To validate the performance and consistency of our design, an optical setup for wide-angle measurements was meticulously executed. The experimental results at the designed focal plane were found to agree with our design predictions. We further conducted an analysis by capturing cross-sectional images along the propagation direction under varying incident angles, discovering light on the physical phenomena associated with wide-angle incidence and the resultant output vortex beam generation. These comprehensive investigations collectively substantiate the efficacy and precision of our wide-angle metasurface approach, paving the way for optical vortex generation and manipulation.

## 2. Metasurface Design and Fabrication

**Figure 1:**
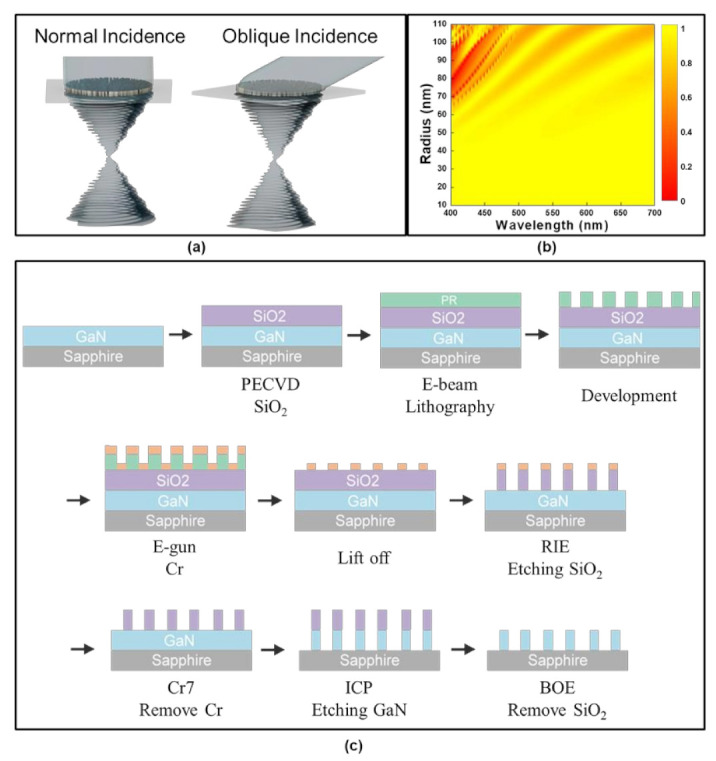
(**a**) Schematic representation illustrating the wide-angle vortex metasurface under normal and oblique incidences; (**b**) Simulated co-polarization transmission efficiency of the wide-angle vortex metasurface as a function of different radii across various wavelengths; (**c**) The fabrication process flow for the metasurface.

Figure 1a schematically illustrates the vortex metasurface proposed herein, detailing its behavior under normal and oblique incidences. The phase retardation distribution of the wide-angle metasurface for vortex beam generation is formulated as follows [21,40]:

(1) $$\varphi \left(x,y\right)=-\frac{2\pi }{\lambda }\left(xsin\left(\alpha_{x}\right)+ysin\left(\alpha_{y}\right)+\sqrt{f^{2}+{\left(x-x_{0}\left(\alpha_{x}\right)\right)}^{2}+{\left(y-y_{0}\left(\alpha_{y}\right)\right)}^{2}}-\sqrt{f^{2}+{\left(x_{0}\left(\alpha_{x}\right)\right)}^{2}+{\left(y_{0}\left(\alpha_{y}\right)\right)}^{2}}\right)+m\times {tan}^{-1}\left(\frac{y}{x}\right)$$

where λ is the operating wavelength, x and y are the Cartesian coordinates, $\alpha_{x}\;\mathrm{and}\;\alpha_{y}$ are the angles of incidence, f is the focal length, $x_{0}\left(\alpha_{x}\right)$ and $y_{0}\left(\alpha_{y}\right)\;$ are the positions of the focal points on the focal plane, and m is the OAM mode and the corresponding topological charge (TC).

In the realm of optical lens design, particularly in the pursuit of wide-angle and fisheye characteristics, several mapping functions have been advanced, including stereographic, equidistant, equisolid angle, and orthographic models. Here, we have adopted the orthographic mapping function to facilitate the realization of our wide-angle metasurface. The selection of this particular mapping function is particularly advantageous for scenarios involving wide-angle or fisheye lenses, where emphasis is placed on peripheral details over central vision. This choice mitigates image compression at the image center. When implementing the orthographic mapping function with $x_{0}\left(\alpha_{x}\right)=fsin\left(\alpha_{x}\right)$ and $y_{0}\left(\alpha_{y}\right)=fsin\left(\alpha_{y}\right)$, the resulting phase distribution takes the form:

(2) $$\varphi \left(x,y\right)=-\frac{2\pi }{\lambda }\left(xsin\left(\alpha_{x}\right)+ysin\left(\alpha_{y}\right)+\sqrt{f^{2}+{\left(x-fsin\left(\alpha_{x}\right)\right)}^{2}+{\left(y-fsin\left(\alpha_{y}\right)\right)}^{2}}-\sqrt{f^{2}+{\left(fsin(\alpha_{x})\right)}^{2}+{\left(fsin(\alpha_{y})\right)}^{2}}\right)+m\times {tan}^{-1}\left(\frac{y}{x}\right)$$

To realize a phase profile independent of incident angles, approximations $sin\left(\alpha_{x}\right)=\frac{x}{2f}$ and $\;sin\left(\alpha_{y}\right)=\frac{y}{2f}$ are applied [40]. This leads us to a phase distribution devoid of incident angle dependence as follows:

(3) $$\varphi \left(x,y\right)=-\frac{\pi }{f\lambda }\left(x^{2}+y^{2}\right)+m\times {tan}^{-1}\left(\frac{y}{x}\right)$$

The phase distribution is implemented through the propagation phase method, chosen for its precise capability to achieve full phase control via meta-structure geometry adjustments. Specifically, the diameters of cylindrical pillars are tailored to attain a 2π phase control, yielding optimal co-polarization transmission efficiency by a commercialized CST tool, as demonstrated in Figure 1b. The radii employed for constructing the 100-µm-diameter metasurface span from 28 to 70 nm with a pillar height of 800 nm while adhering to a subwavelength period of 220 nm at the designed wavelength of 450 nm, resulting in an average co-polarization transmission efficiency simulated as high as 97%. This metasurface configuration entails other parameters, including an OAM mode of 12 and a focal length of 45 µm.

The metasurface fabrication entails a sequential procedure, as shown in Figure 1c. Commencing with an 800-nm-thick, un-doped GaN epi-layer on a double-polished sapphire substrate, a subsequent step involves depositing a 400-nm-thick silicon dioxide (SiO_2_) layer as a hard mask on this foundation. Utilizing an electron-beam lithography system, the requisite patterns are defined via direct exposure of a concentrated electron beam onto the top surface of the SiO_2_-deposited substrate, which features an electron-resist coating with a positive resist ZEP-520A. The process encompasses the evaporation of a 30-nm-thick chromium (Cr) layer, serving as a hard mask. Subsequent to this evaporation, and in order to achieve the desired outcome, a lift-off process is executed on the sample. The subsequent stages entail the utilization of dry etching methodologies involving both reactive-ion etching (RIE) and inductively coupled plasma reactive-ion etching (ICP-RIE) systems. The ICP-RIE system operated at a radio frequency (RF) of 13.56 MHz, employing an ICP source power of 700 W and a bias power of 280 W while utilizing a BCl_3_/Cl_2_ chemistry mixture. The etching rate of GaN achieved through ICP-RIE in this study was measured at a rate of 13 nm per second. The residual SiO_2_ hard mask layer was eliminated by immersing the sample into a Buffered Oxide Etch (BOE) solution. These processes are adeptly employed to fabricate the high-aspect-ratio meta-structures comprised of GaN. The fabrication processes for high-aspect-ratio GaN structures demand precise parameter tuning within the ICP-RIE equipment. Deviations in device performance, such as undesirable slanted sidewall morphology or low aspect ratios of GaN meta-structures, were observed to lead to discrepancies from the optimally simulated co-polarization transmission efficiency, which typically reached an average value of up to 97%. These deviations also manifested in variations between the measured focal length and the intended design value.

**Figure 2:**
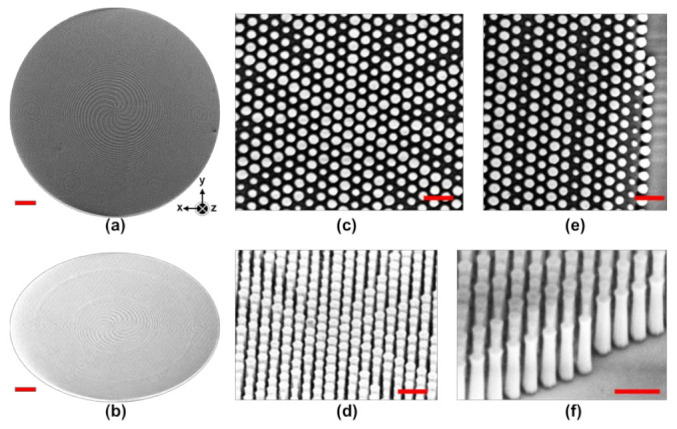
The SEM images of the fabricated metasurface from the (**a**) top view and (**b**) tilt view; Zoomed-in top-view SEM images taken at the (**c**) center and (**e**) edge regions; Magnified tilt-view SEM images taken at the (**d**) center and (**f**) edge regions; Scale bar: 10 µm in (**a**,**b**); Scale bar: 500 nm in (**c**–**f**).

Figure 2a,b show SEM images capturing both the top and tilt views of the fabricated metasurface. These images distinctly reveal the discernible spiral branches inherent to the metasurface design, and the count of observed spiral arms aligns precisely with our meticulously devised configuration. Detailed insights are further elucidated in Figure 2c,e, which provide magnified top-view SEM images taken at both the center and edge regions. Corresponding tilt-view counterparts are thoughtfully presented in Figure 2d,f, collectively highlighting the successful realization of high-aspect-ratio metasurfaces fabricated from the wide-bandgap material GaN. It is necessary to emphasize the significant challenge associated with achieving such vertically inclined meta-structures while simultaneously preserving the exceptional smoothness and precision of their sidewalls.

## 3. Experimental Results of the Metasurface

**Figure 3:**
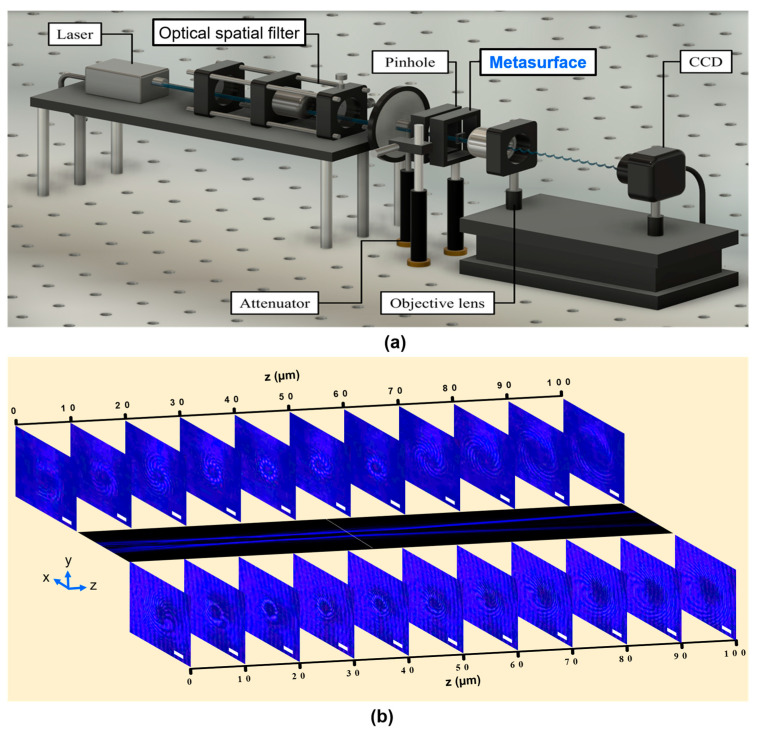
(**a**) Schematic representation illustrating the optical setup designed for measuring the donut intensity profiles under normal illumination; (**b**) Central panel: the measured intensity distribution in the x-z plane; the interference measurements superimposing the reference beam carrying the spherical wavefront (**Top panel**) or the planar wavefront (**Bottom panel**) at different *z*-axis positions.

To validate the convergence of the metasurface at the intended focal distance, it is imperative to execute the optical configuration depicted in Figure 3a. The assessment enables an exploration of the donut intensity distribution exhibited by the device. In this experimental setup, the metasurface is subjected to illumination by the incident light generated through a laser beam operating at a wavelength of 450 nm. This incident light traverses a spatial filter and a pinhole characterized by a diameter of 25 µm. Subsequent to its interaction with the metasurface, the emergent light is collected by an objective lens and subsequently directed to a charge-coupled device (CCD) for recording. The resulting intensity distribution within the x-z plane for the wide-angle configuration, which embodies an OAM mode of 12, is illustrated in Figure 3b (center panel). The most diminutive diameter of the observed donut pattern manifests at the designated distance of 45 µm, accentuated by the white-dashed line within the figure.

Furthermore, an inherent characteristic of vortex beams lies in the emergence of spiral branches observable via the Mach–Zehnder interferometer. Our measurement setup closely resembles that depicted in Figure 3a, with the following modification, the incident light beam was split into signal and reference beams to facilitate the necessary interference. This interference phenomenon is meticulously captured and subsequently presented in Figure 3b, where the resulting patterns in the x-y plane are shown at various *z*-axis distances. The upper and lower depictions within the figure correspond to the interference outcomes stemming from the reference beam with spherical and planar wavefronts, respectively.

The visual representations within the figures reveal an intriguing behavior: the helicity of the spiral branches within the x-y plane preserves a clockwise orientation until a distinctive petal-like pattern appears at a *z*-axis position of 40 µm, diverging from the intended focal distance of 45 µm. Subsequent to the emergence of this petal-like configuration, a notable shift in the helicity of the spiral pattern occurs, transitioning into a counterclockwise rotation. This noteworthy transformation is attributed to the strategic alteration in the helicity of the spiral branches, a direct consequence of the emergence of the petal-like pattern at a strategically significant location. This alteration serves the purpose of attaining the most diminutive donut diameter precisely at the designated focal length.

**Figure 4:**
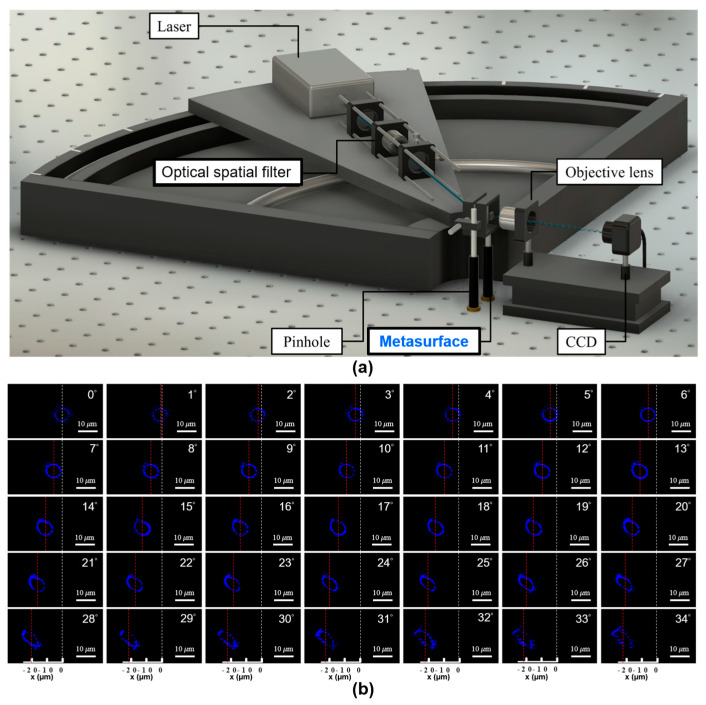
(**a**) Schematic illustration of the optical setup devised for wide−angle measurement; (**b**) Donut intensity distributions for incident angles ranging from 0 to 34 degrees, where 0 degrees represents normal incidence. White−dashed lines correspond to the donut’s center under normal incidence. Red-dashed lines distinctly indicate the center for incident angles other than zero degrees.

The investigation into the wide-angle capabilities of the device is conducted through a meticulously designed experimental setup, illustrated in Figure 4a. Our study encompasses the analysis of the donut intensity distributions in the x-y plane, precisely at the focal distance of 45 µm. The intriguing feature of this part lies in the distinct range of incident angles explored, spanning from 0 to 34 degrees (with 0 degrees denoting normal incidence), as depicted in Figure 4b. The white-dashed lines correspond to the donut’s center under normal incidence, while the red-dashed lines distinctly denote the center for incident angles other than zero degrees. Clearly evident in the captured images are the intensity distributions of a donut shape, displaying pronounced characteristics at incident angles of up to 30 degrees. Beyond this range, the completeness of the shape gradually diminishes. With an augmented incident angle, the positions of the donut perceptibly shift towards greater *x*-axis coordinates in the negative direction.

**Figure 5:**
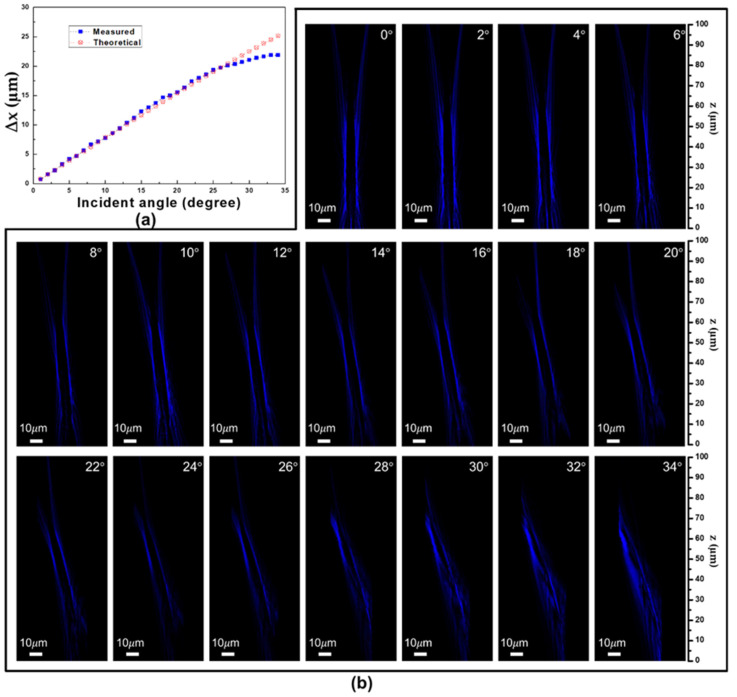
(**a**) The difference in measured central positions of the donut between various incident angles relative to the zero-degree normal incidence; (**b**) Metasurface’s intensity distributions in the x-z plane for incident angles spanning from 0 to 34 degrees.

As illustrated in the figures, the disparity in measured central positions of the donut between the zero-degree incident angle and incident angles ranging from 1 to 34 degrees—incrementing by 1 degree each—closely resembles a set of corresponding theoretical values (∆x = f sin(θ)). The comparison between measured and theoretical values can be found in Figure 5a, corresponding to incident angles spanning from 1 to 34 degrees. Alignment is observed between these theoretical values and the experimental findings up to the incident angle of 30 degrees. This observation distinctly underscores the exceptional effectiveness of the fabricated metasurfaces, providing unequivocal evidence of their superior performance. However, upon surpassing the 30-degree threshold, the measured values deviate from the corresponding theoretical predictions, manifesting a plateau-like trend in proximity to the 21 µm point. This behavior aligns with the trends elucidated in Figure 4b, wherein the donut-shaped structure progressively loses its completeness.

Consequently, our analysis was extended to encompass intensity distributions in the x-z plane. This investigation spanned incident angles ranging from 0 to 34 degrees, in increments of 2 degrees, as vividly depicted in Figure 5b. As incident angles increase, discernible tilting behavior becomes apparent within the measured distributions. Notably, beyond an incident angle of 30 degrees, these distributions take on an asymmetric nature, yielding an emergence of undesirable light components. These outcomes are consistent with the observations presented in Figure 4b and Figure 5a, reinforcing the metasurface’s exceptional capabilities within incident angles of up to 30 degrees. The metasurface’s performance is robustly evaluated through consistent examination of intensity distributions in both the x-y and x-z planes. The metasurface with the smallest focal spots, characterized by a symmetrical donut profile and positioned precisely at the designated *z*-axis position of 45 µm, manifests exclusively under diverse oblique incidences when the metasurface maintains a symmetrical intensity distribution in the x-y plane. Elevating the angle of oblique incidence necessitates an enhanced design methodology specifically tailored for wide-angle metasurface realization. Crucially, the realization of metasurfaces with large-angle oblique incidences requires the construction of high-aspect ratio GaN meta-structures proposed in this study.

## 4. Summary

In this study, we have successfully incorporated wide-angle capability into vortex beams with the exceptionally high TC of 12 through the utilization of a meticulously engineered propagation-phase-designed metasurface. Comprising high-aspect-ratio GaN meta-structures, this metasurface demonstrates an exceptional average co-polarization transmission efficiency, reaching a simulated value as high as 97%. The intricate spiral patterns, along with their respective count numbers, have undergone meticulous investigation through tilt-view SEM and further elucidation via the Mach–Zehnder interferometer. An intriguing observation reveals a petal-like interference pattern manifesting prior to the metasurface’s design focal distance. The emergence of this petal-like pattern at a specific *z*-axis position serves as a threshold, prompting a strategic alteration in the helicity of the spiral branches. This strategic change in helicity is directly linked to achieving a minimized donut diameter at the designated focal length. Regarding the wide-angle capability of the device, captured images unmistakably showcase intensity distributions in the form of donut shapes in the x-y plane. These distributions distinctly manifest pronounced characteristics within incident angles of up to 30 degrees. Nevertheless, as incident angles exceed the 30-degree mark, the measured values diverge from their corresponding theoretical predictions, resulting in a gradual loss of completeness within the donut-shaped structure. The metasurface’s performance is robustly evaluated through consistent examination of intensity distributions in both the x-y and x-z planes.

## Data Availability

The data regarding the findings of this study are available from the corresponding author upon reasonable request.
